# Dance- and movement-based interventions for autism spectrum disorder: a systematic review and exploratory meta-analysis

**DOI:** 10.3389/fpsyt.2026.1994047

**Published:** 2026-10-28

**Authors:** Xiangyu Meng, Zhixi Xiang, Jun Wang

**Affiliations:** 1 School of Music and Dance, South-Central Minzu University, Wuhan, China; 2 School of Public Health, Tongji Medical College, Huazhong University of Science and Technology, Wuhan, China; 3 School of Life Science and Health Engineering, Hubei University of Technology, Wuhan, Hubei, China

**Keywords:** autism spectrum disorder, dance- and movement-based interventions, dance/movement therapy, motor function, social communication

## Abstract

**Objectives:**

Autism spectrum disorder (ASD) is a heterogeneous neurodevelopmental condition characterized by difficulties in social communication, restricted and repetitive behaviors, and impaired adaptive functioning. Although behavioral and developmental interventions remain central to ASD management, the effects of dance- and movement-based interventions (DMIs) across different functional domains have not been fully clarified. This study aimed to systematically evaluate the effects of DMIs on overall autism symptom severity, social communication/social responsiveness, and motor function in individuals with ASD.

**Methods:**

This systematic review and meta-analysis was conducted following the Preferred Reporting Items for Systematic Reviews and Meta-Analyses (PRISMA) 2020 guidelines. Eligible DMIs included formal dance/movement therapy or dance movement psychotherapy and other structured dance- or purposeful movement programs in which movement constituted a central therapeutic or rehabilitative component. PubMed, Web of Science, Embase, the Cochrane Library, and PsycINFO were searched from inception to August 2026. Randomized and non-randomized controlled studies were not combined in the same primary meta-analysis. Randomized studies reporting sufficiently comparable outcomes were synthesized using standardized mean differences (SMDs) with 95% confidence intervals (CIs) under random-effects models. Risk of bias was assessed using RoB 2 and ROBINS-I, and certainty of evidence was evaluated using GRADE.

**Results:**

Twelve controlled studies involving individuals with ASD were included and showed substantial variation in intervention format, comparator conditions, outcome measures, and risk of bias. Exploratory RCT-only meta-analyses included three studies for social communication/social responsiveness (n = 76; SMD = −0.68, 95% CI: −1.58 to 0.23; p = 0.15; I² = 72%) and three studies for motor function (n = 55; SMD = 2.04, 95% CI: −0.52 to 4.61; p = 0.12; I² = 92%); certainty of evidence was very low for both domains. Overall autism symptom severity was represented by one randomized trial (n = 30; SMD = −3.09, 95% CI: −4.19 to −1.99), resulting in low-certainty evidence and precluding pooling. Narrative synthesis identified potentially favorable signals in joint engagement, cooperative behavior, interpersonal synchrony, emotion inference, and gesture imitation, but these findings arose from small and heterogeneous studies.

**Conclusion:**

The current evidence base for DMIs is small, heterogeneous, and of low to very low certainty. Structured narrative synthesis suggests possible domain-specific benefits, whereas exploratory pooled estimates for social communication/social responsiveness and motor function remain inconclusive. DMIs may have potential as complementary interventions for ASD, but larger, methodologically rigorous randomized trials with standardized interventions and outcome measures are required.

**Systematic review registration:**

https://www.crd.york.ac.uk/PROSPERO/view/CRD420261495230, identifier CRD420261495230.

## Graphical Abstract

**Figure:**
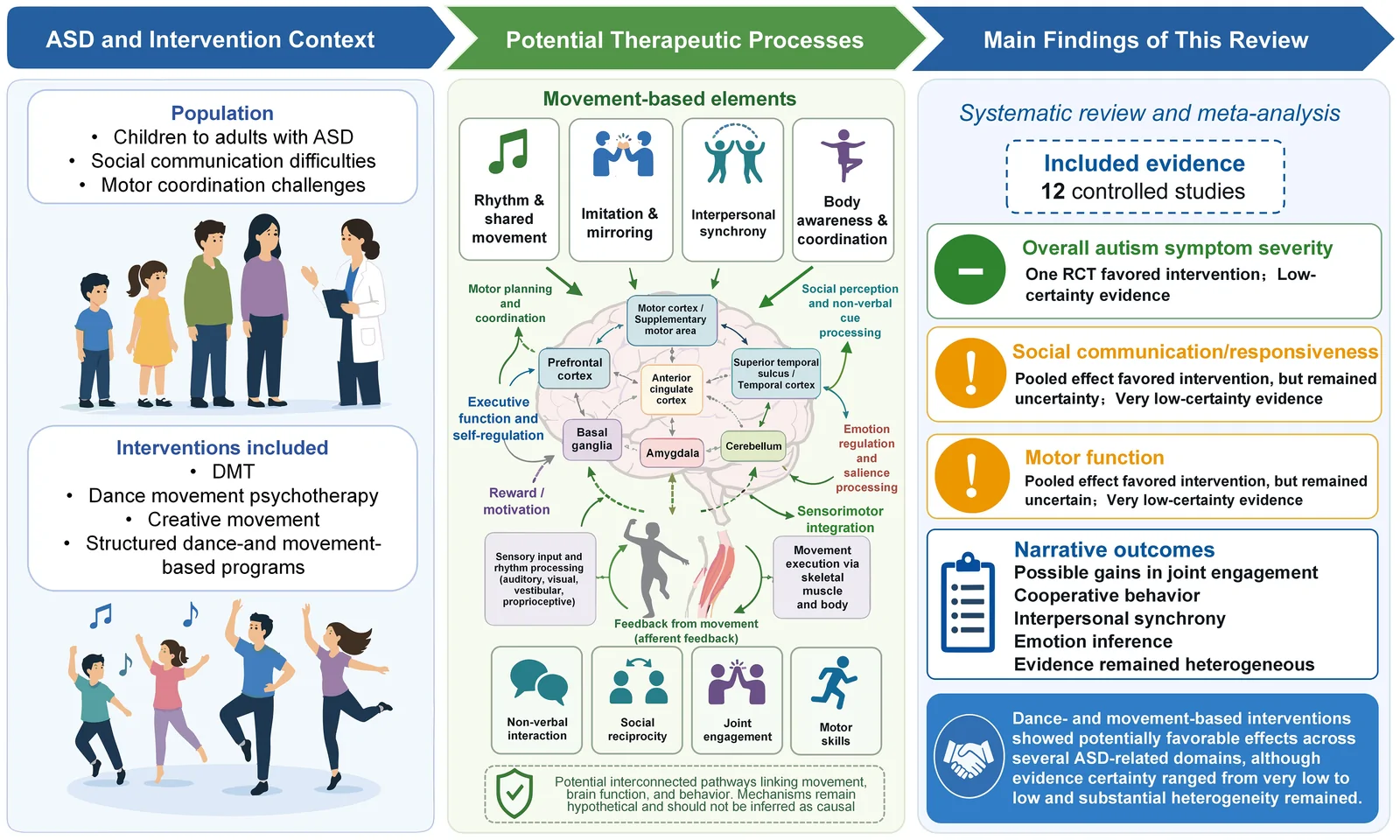
Overview of the intervention spectrum, principal outcome domains, and certainty of evidence for dance- and movement-based interventions in autism spectrum disorder.

Provide the Systematic Review Registration details (Unique Identifier and URL of the publicly accessible website), if applicable.

## 1 Introduction

Autism spectrum disorder (ASD) is a heterogeneous neurodevelopmental condition characterized by persistent differences in social communication and reciprocal social interaction, together with restricted and repetitive patterns of behavior, interests, or activities (1, 2). Its presentation varies widely across individuals and across development, and the effects on daily life are equally variable. Current epidemiological estimates also point to a substantial public-health burden. The U.S. Autism and Developmental Disabilities Monitoring Network identified ASD in 1 in 31 8-year-old children in 2022 (3), while the Global Burden of Disease Study 2021 estimated that 61.8 million people worldwide, or about 1 in 127, were on the autism spectrum (4). In China, a nationwide study reported a prevalence of 0.70% among children aged 6–12 years (5). The social and economic burden of ASD is substantial, including heightened family stress, reduced quality of life, and high costs of long-term care and educational support. These estimates differ across populations and surveillance methods, but they underscore the scale of ASD and the need to address the functional difficulties that accompany it.

Motor difficulties are one of these less visible but clinically relevant areas. They are not part of the core diagnostic criteria, yet systematic reviews and meta-analyses have documented differences in motor coordination, postural control, gross and fine motor skills, and early motor development (6–8). Such difficulties can affect play, physical activity, learning, and participation in everyday social situations. They may also intersect with social functioning. Most studies included in one systematic review reported an association between motor and social skills in ASD (9), and atypical interpersonal motor synchrony has been described in social interaction (10). For intervention research, motor outcomes therefore merit separate attention rather than being treated only as secondary behavioral measures.

Medication played a limited role in treating the core features of ASD and was used mainly for associated symptoms or co-occurring conditions (11, 12). Behavioral, developmental, communication, and social interventions remained central to care and could improve selected outcomes, although response varied considerably (2, 13). Some behavioral programs required intensive practice, sustained therapist or family involvement, or highly structured teaching (13). For minimally verbal individuals, communication interventions that did not depend solely on spoken language, including augmentative and alternative communication approaches, were also investigated as alternatives or complements (14). These differences in treatment demands prompted interest in complementary approaches that could engage participants through non-verbal interaction while addressing social and motor processes within the same activity.

For the purposes of this review, dance- and movement-based interventions (DMIs) were defined as a broad category of structured interventions in which dance or purposeful movement formed a central therapeutic or rehabilitative component, regardless of whether the program was delivered within a formal psychotherapy framework. Within this broader category, dance/movement therapy (DMT)—also termed dance movement psychotherapy (DMP) in some settings—is a formal psychotherapeutic approach in which movement is used to support emotional, social, cognitive, and physical integration (15, 16). Other DMIs evaluated in ASD included creative movement, inclusive community dance, and imitation- or synchronization-based programs (17–19). Across these formats, rhythm, body awareness, imitation, mirroring, and shared movement provided opportunities for participants to respond to another person’s actions and coordinate movement in real time (20). Action-observation processes and embodied accounts of cognition have been proposed as possible explanations for such effects (21, 22), although the mechanisms of clinical change remain uncertain. The diversity of the field was reflected in controlled studies ranging from imitation- and synchronization-based work with autistic adults (17) to protocol-based DMP in children (18) and inclusive community dance programs (19).

Despite the growing number of studies, the evidence remained difficult to compare. Earlier reviews reported possible benefits in social reciprocity, communication, body awareness, emotional expression, and sensorimotor functioning, but repeatedly noted small samples, heterogeneous interventions, and variable methodological quality (16, 23, 24). A 2022 meta-analysis of seven controlled studies found pooled effects favoring dance interventions for overall ASD symptoms and social interaction, but not empathy (25). Reviews of physical activity more broadly have also reported improvements in social communication, although they combine exercise modalities that are not directly comparable with DMT or dance-based programs (26). A 2026 meta-analysis of 14 randomized trials in children and adolescents reported favorable effects on several social and behavioral outcomes (27), but its search ended in March 2025, it excluded adults and non-randomized controlled designs, and motor function was not analyzed as a separate pooled domain. Evidence published after that search further broadened the field: one randomized trial examined synchronized group movement and prosocial behavior in young autistic adults (28), while a longer-term controlled DMT study followed children for 11 months and assessed global symptoms, repetitive behaviors, and social responsiveness (29). The main uncertainty is therefore no longer simply whether dance-related interventions can produce change, but whether effects are consistent across age groups and study designs, whether motor function shows a distinct intervention effect, and which additional outcomes can be meaningfully combined. The number of randomized studies identified in the present review was smaller than that reported in the recent meta-analysis by Jingyao et al. (27), despite the later search date and broader age eligibility. This difference primarily reflects differences in search coverage and intervention eligibility rather than the search period alone. Jingyao et al. searched several Chinese databases, including CNKI, Wanfang, and VIP, and included a broad spectrum of dance activities, including standardized dance training and several Chinese-language or locally indexed studies. In contrast, the present review searched five international bibliographic databases and required dance or purposeful movement to constitute a predefined central therapeutic or rehabilitative component; general exercise, sports training, and recreational activities without a substantive dance/movement component were excluded. Accordingly, the two reviews captured partially different evidence bases. The present review extends the earlier synthesis by including adults and non-randomized controlled studies in the systematic review, separating randomized from non-randomized evidence in quantitative synthesis, evaluating motor function as a distinct outcome domain, incorporating studies published after the earlier search, and applying design-specific risk-of-bias assessment and GRADE.

We therefore conducted a systematic review and meta-analysis of controlled studies involving individuals with ASD across age groups and intervention formats. Eligible interventions included formal DMT and other structured dance- or movement-based programs, with usual care, wait-list, no-intervention conditions, and active comparators considered as controls. Given the limited number of randomized trials available in this field, we included controlled studies with different designs to characterize the current evidence base. Outcomes were organized into three prespecified domains: overall autism symptom severity, social communication/social responsiveness, and motor function. Quantitative pooling was undertaken when at least two randomized studies assessed sufficiently comparable outcomes; otherwise, findings were synthesized narratively and, where appropriate, individual study effect estimates were reported. Outcomes that were insufficiently comparable for quantitative pooling—including empathy and emotion inference, joint engagement, cooperative behavior, interpersonal synchrony, sensory and emotional measures, verbal and non-verbal communication, and neurophysiological outcomes—were summarized narratively. By examining these domains separately, we aimed to clarify where evidence is relatively consistent and where interpretation remains constrained by heterogeneity. This domain-specific synthesis may help clinicians interpret the current evidence more cautiously and guide researchers in developing future trials with standardized interventions and outcome measures.

## 2 Materials and methods

This systematic review and meta-analysis was conducted in accordance with the Preferred Reporting Items for Systematic Reviews and Meta-Analyses (PRISMA) 2020 statement (30). The review protocol was registered retrospectively in the International Prospective Register of Systematic Reviews (PROSPERO; CRD420261495230) (31).

### 2.1 Search strategy

A systematic search of PubMed, Web of Science, the Cochrane Library, Embase, and PsycINFO was conducted from database inception to 1 August 2026. Search terms combined concepts related to autism spectrum disorder and dance- or movement-based interventions. The core search strategy was: (Autis* OR “Autism Spectrum Disorder” OR Asperger* OR “Pervasive Developmental Disorder”) AND (“Dance Therapy” OR “Dance Movement Therapy” OR “Dance/Movement Therapy” OR “Dance Movement Psychotherapy” OR “Movement Therapy” OR “Creative Movement Therapy” OR “Dance Intervention” OR “Dance Activity”). Search syntax was adapted to each database. No language or publication-date restrictions were applied. Search reporting was guided by PRISMA-S recommendations (32).

For each search, titles and abstracts were screened, and full-text versions of articles meeting the criteria were downloaded. The full texts were reviewed, and any reference articles not yet obtained were ordered and acquired.

### 2.2 Eligibility criteria

Studies were eligible for inclusion in the systematic review if they met all of the following criteria: (1) participants had a diagnosis of ASD or an autism-spectrum condition established according to internationally recognized diagnostic criteria, including DSM-IV, DSM-5, ICD-10, or other validated diagnostic procedures reported by the original study (33); (2) the intervention consisted of DMT, dance movement psychotherapy, creative movement, or another structured dance- or movement-based program in which intentional movement, expressive movement, rhythm, or interpersonal movement interaction represented a central therapeutic component. Interventions primarily involving general physical exercise, sports training, or recreational activities without a defined dance/movement component were excluded; combined interventions were eligible when the dance/movement component was predefined, distinguishable from other components, and accounted for a meaningful proportion of the intervention content; (3) the study used a controlled interventional design, including randomized controlled trials, randomized crossover trials, wait-list controlled trials, quasi-experimental studies, or other prospective controlled designs, with a concurrent comparator; (4) the study reported measurable quantitative outcomes related to autism symptom severity, social or communicative functioning, motor performance, psychosocial functioning, or intervention-related neurophysiological changes.

Studies were excluded if they met any of the following criteria: (1) participants did not have ASD, or ASD-specific data could not be separated from a mixed diagnostic sample; (2) dance or structured movement was not a substantive component of the intervention; (3) no clearly defined concurrent control or comparator group was included; (4) the report was a review, protocol, purely qualitative study, case report, uncontrolled case series, or conference abstract/other report with insufficient methodological or outcome information; or (5) the same or substantially overlapping participant sample was reported in more than one publication, in which case the report providing the most complete data for the relevant outcome was retained and related reports were not treated as independent studies.

For quantitative synthesis, studies were additionally required to provide sufficient numerical data for effect-size estimation and to report outcomes that were sufficiently comparable in both clinical construct and measurement domain. Randomized and non-randomized studies were not combined in the same primary meta-analysis. Where at least two randomized studies assessed sufficiently comparable outcomes, randomized studies were pooled using meta-analysis. Non-randomized controlled studies and outcomes that were not considered sufficiently comparable for pooling were retained in the systematic review and synthesized narratively.

### 2.3 Data extraction

Two authors (M.X. and X.X.) independently screened the literature, reviewed titles and abstracts, and assessed full-text articles for eligibility. Any disagreement regarding study inclusion was resolved through discussion and consensus, with adjudication by a third author (W.J.) when necessary. Reasons for exclusion at the full-text stage were recorded, and the study-selection process was summarized using a PRISMA flow diagram.

Data were extracted using a standardized form. Extracted items included first author, publication year, country or region, study design, sample size, sex distribution, participant age, intervention and comparator characteristics, intervention duration, outcome measures, and quantitative outcome data. For continuous outcomes, post-intervention means, standard deviations (SDs), and sample sizes were extracted. The quantitative data used in the meta-analyses were checked against the original reports. Particular attention was given to studies producing unusually large, standardized effect estimates to verify whether the extracted values represented SDs rather than SEs and whether endpoint values, rather than change scores or adjusted estimates, had been entered.

Outcomes were grouped according to conceptual similarity into overall autism symptom severity, social communication/social responsiveness, motor function, and other outcomes. When more than one measure within a study assessed the same domain, one measure was selected according to conceptual relevance and comparability with measures used in the other included studies, so that each study contributed no more than one effect estimate to a given meta-analysis. Outcomes that were not sufficiently comparable for quantitative pooling, including empathy and emotion inference, joint engagement, cooperative behavior, interpersonal synchrony, sensory and emotional measures, verbal and non-verbal communication, and neurophysiological outcomes such as functional near-infrared spectroscopy (fNIRS)-derived measures (34), were synthesized narratively.

### 2.4 Risk-of-bias assessment

Risk of bias was assessed separately according to study design. Randomized studies were evaluated using the Cochrane Risk of Bias 2 (RoB 2) tool, whereas non-randomized controlled intervention studies were assessed using the Risk Of Bias In Non-randomized Studies of Interventions (ROBINS-I) tool (35).

For RoB 2, risk of bias was assessed across five domains: bias arising from the randomization process, bias due to deviations from intended interventions, bias due to missing outcome data, bias in measurement of the outcome, and bias in selection of the reported result. Overall judgments were categorized as low risk, some concerns, or high risk. For ROBINS-I, the domains included bias due to confounding, selection of participants, classification of interventions, deviations from intended interventions, missing data, measurement of outcomes, and selection of the reported result. Overall judgments were categorized according to the ROBINS-I framework (35).

Risk-of-bias assessments were performed at the outcome level where appropriate. Judgments were based on information reported in the original studies, and disagreements were resolved through discussion and consensus.

### 2.5 Evidence synthesis and statistical analysis

All included studies were first synthesized narratively according to study design, participant characteristics, intervention components, comparator conditions, outcome measures, and risk-of-bias judgments. Meta-analysis was used as a secondary, exploratory synthesis when at least two randomized studies reported outcomes judged sufficiently comparable in clinical construct and measurement domain. Non-randomized controlled studies were not pooled with randomized trials and were retained in the narrative synthesis. Meta-analyses were performed in Review Manager (RevMan), version 5.4.1.

Because conceptually related outcomes were assessed using different instruments, effect sizes were expressed as standardized mean differences (SMDs) with 95% confidence intervals (CIs). The SMD was calculated as Hedges’ adjusted g, using the pooled within-study SD and a small-sample correction (36). Post-intervention endpoint values were used, and random-effects models were applied because clinical and methodological variation across studies was anticipated (37).

For crossover trials, first-period data before crossover were used when available to avoid double counting and potential carryover effects (38); this approach was applied to Aithal et al. For multi-arm studies, only groups relevant to the comparison were entered, without duplicating participants within a meta-analysis.

Before pooling, the direction and conceptual meaning of each outcome measure were examined. For social communication/social responsiveness outcomes, higher scores indicated greater impairment, and negative SMDs therefore favored the intervention. For motor outcomes, higher scores were aligned to indicate better performance, such that positive SMDs favored the intervention. Differences in the constructs represented by the individual instruments were also considered when determining whether outcomes were sufficiently comparable for quantitative synthesis.

The social communication/social responsiveness meta-analysis included randomized studies assessing the SCQ, SRS-2 Social Communication and Interaction score, or ATEC social ability domain. The motor-function meta-analysis included randomized studies assessing gross motor performance, MABC-2 performance, or objective motor coordination. Because each individual instrument was represented by only one study, instrument-specific subgroup analyses were not considered statistically appropriate. After restriction to randomized evidence, only one randomized trial contributed data on overall autism symptom severity; therefore, a pooled meta-analysis was not performed for this domain, and the evidence was summarized narratively together with the individual study effect estimate.

Statistical heterogeneity was assessed using Cochran’s Q test and quantified with the I² statistic (39). I² values of approximately 0%-40%, 30%-60%, 50%-90%, and 75%-100% were interpreted as potentially unimportant, moderate, substantial, and considerable heterogeneity, respectively, while also considering the magnitude and direction of effects. Because considerable heterogeneity was observed in the motor-function analysis, leave-one-out sensitivity analyses were performed by sequentially excluding each study to evaluate the stability of the pooled estimate.

A two-sided *p* value < 0.05 was considered statistically significant. Publication bias was not formally assessed because fewer than 10 studies contributed to each meta-analysis, making funnel-plot asymmetry tests unreliable in this setting (40).

### 2.6 Certainty of evidence

The certainty of evidence for the main outcome domains was assessed using the Grading of Recommendations Assessment, Development and Evaluation (GRADE) approach (35). Evidence was evaluated across the domains of risk of bias, inconsistency, indirectness, imprecision, and publication bias. Certainty was categorized as high, moderate, low, or very low. Summary of Findings tables were generated using GRADEpro GDT for social communication/social responsiveness, motor function, and overall autism symptom severity.

## 3 Results

### 3.1 Study selection and structured narrative synthesis of included studies

A systematic literature search identified a total of 963 records from PubMed (n = 412), Web of Science (n = 276), Cochrane Library (n = 183), Embase (n = 59), and PsycINFO (n = 33). After removing duplicates, 780 records remained for title and abstract screening. Of these, 728 records were excluded based on title and abstract review, and 52 reports were sought for retrieval. Twelve reports could not be retrieved, leaving 40 full-text reports for eligibility assessment. Of these, 28 were excluded because they were case studies (n = 3), non-intervention studies (n = 2), did not involve an eligible dance- or movement-based intervention (n = 3), included an ineligible study population (n = 4), or did not provide sufficient quantitative outcome information to meet the eligibility criteria (n = 16). Ultimately, 12 studies met the eligibility criteria and were included in the systematic review (**Figure 1**).

**Figure 1:**
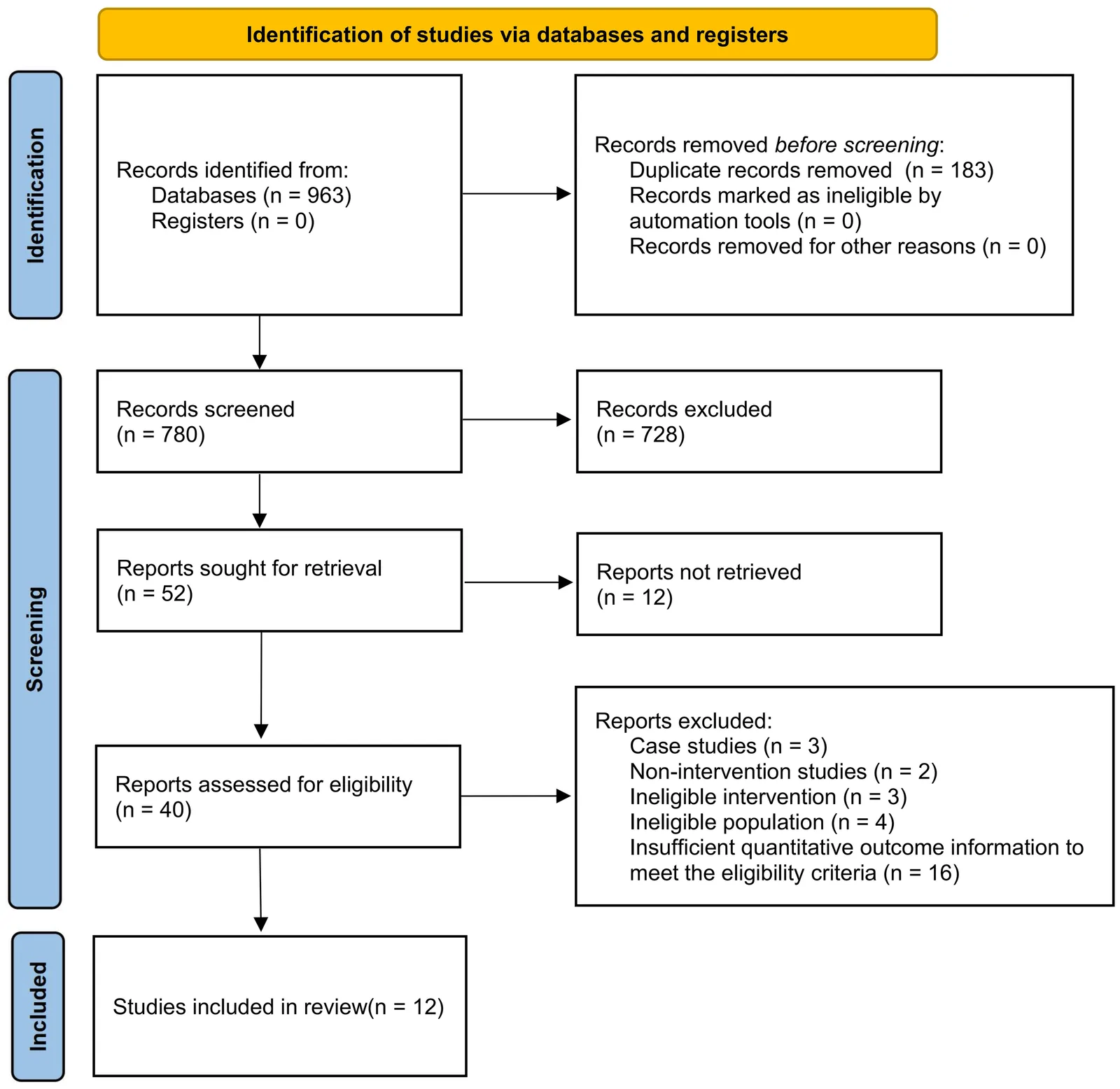
PRISMA flow diagram of study identification, screening, eligibility assessment, and inclusion. The diagram summarizes the number of records identified from each database, records remaining after duplicate removal, articles screened at the title/abstract and full-text stages, reasons for full-text exclusion, and the final number of studies included in the systematic review.

The 12 included studies comprised eight randomized studies and four non-randomized controlled studies and included children, adolescents, and adults with ASD. Sample sizes were generally small, ranging from 14 to 57 participants, and intervention duration ranged from approximately 5 weeks to 1 year. Considerable variation was evident in participant age, study design, intervention format, comparator condition, treatment duration, and outcome assessment (**Table 1**).

**Table 1:** Characteristics of the studies included in the systematic review.

Study	Country / region	Study design	Samplesize		Age(year)		Intervention / Comparator	Duration	Outcomes /main findings
			(INT) (males/females)	(Ctrl) (males/females)	INT	Ctrl			
Aithal_2021	UK	Randomized crossover pilot	26 21/5	26 21/5	10.65 ± 1.67		Protocol-based DMP / Standard care	5 weeks	SCQ, SDQ; SCQ improved, SDQ not significant
Chiang_2016	Taiwan	Quasi-experimental	18	16	2.99±0.72	3.29±0.72	Caregiver-mediated joint engagement +creative body movement play / Control group	10 weeks	Joint engagement; coordinated engagement improved
Cui_2024	China	RCT	12 (12/0)	12 (12/0)	8.08±1.97	8.25±1.28	DMI / Control group	12 weeks	Motor function, SRS; favorable motor and social changes
Dvir_2025	Israel	RCT	26 (25/1)	28 (25/3)	19.51±0.87	19.42±0.83	Synchronized movement / Non-synchronized movement	6-7 weeks	Cooperation, social closeness, stress; favorable cooperation/closeness
Koehne_2016	Germany	Non-randomized controlled	27 (18/9)	24 (14/10)	33.5±9.10	32.0±9.10	Dance therapy / Control movement	10 weeks	Emotion inference, empathy, synchrony; emotion inference/synchrony improved
Lindor_2023	Australia	Pilot RCT	14 (7/7)	13 (6/7)	9.00±1.47	9.08±1.44	AllPlay Dance / Wait-list control	10 weeks	MABC-2, SRS-2, psychosocial outcomes; favorable motor trends
Ma_2025	China	Non-randomized controlled	17 (12/5)	11 (10/1)	13.29±1.80	13.18±1.60	DMT / Rehabilitation training	11 months	ABC, RBS-R, SRS, SCQ; favorable symptom/social changes
Mastrominico _2018	Germany	RCT	35 (27/8)	22 (17/5)	22.5±8.5		Mirroring-based manualized DMT / Wait-list control	10 weeks	Empathy; no significant overall effect
Mateos-Moreno & Lidia Atencia_Doña _2013	Spain	Non-randomized controlled	8 (7/1)	8 (8/0)	25.0 ± 4.10	25.62 ± 8.05	DMT+music therapy / Control	17 weeks	ECA-R; improvements in selected symptom domains
Ren_2022	China	RCT	15	15	5-8		Parent–child MT+DMT / No intervention	3 months	CARS, ATEC; favorable symptom/social changes
Souissi_2026	Tunisia	RCT	7 (7/0)	7 (7/0)	10.29±1.50		Psychomotor DMT / Control	1 year	Motor coordination, gesture imitation; objective motor outcomes improved
Su_2025	USA	Pilot RCT	CM: 8 (6/2)	GM:7(7/0) SP: 7(6/1)	9.50±0.96(SE)	GM:8.42±0.95(SE) SP:8.56±0.94(SE)	Creative movement (CM) / General movement + sedentary play	8 weeks	IPS, fNIRS; CM showed favorable behavioral/neural changes

Study characteristics include country or region, study design, intervention and control sample sizes, participant age, intervention and comparator characteristics, intervention duration, and reported outcomes or main findings.

The interventions differed substantially in their therapeutic orientation and movement components. Aithal et al. evaluated a protocol-based dance movement psychotherapy (DMP) program against standard care in children with ASD, whereas Mastrominico et al. examined a manualized DMT program emphasizing mirroring against a wait-list control in adults. Ma et al. compared DMT with rehabilitation training, while Mateos-Moreno and Atencia-Doña evaluated an intervention combining DMT with music therapy. Ren et al. used a parent–child intervention incorporating music therapy and DMT, compared with no intervention. These studies therefore differed not only in intervention content but also in the extent to which movement was embedded within a psychotherapeutic, rehabilitative, or multimodal framework.

Other studies used structured movement approaches that were distinct from formal DMT/DMP. Lindor et al. evaluated the AllPlay Dance program against a wait-list condition, whereas Cui et al. examined a structured dance/movement intervention against a control condition. Souissi et al. used a psychomotor dance/movement intervention, with motor coordination and gesture imitation among the principal outcomes. Chiang et al. evaluated caregiver-mediated joint-engagement training incorporating creative body-movement play. Dvir et al. directly compared synchronized with non-synchronized movement, thereby isolating interpersonal synchronization more specifically than studies using passive or wait-list controls. Similarly, Koehne et al. compared dance therapy with a control movement condition and focused on emotion inference, empathy, and interpersonal synchrony. Su et al. compared creative movement with general movement and sedentary play and additionally incorporated behavioral and fNIRS-derived outcomes. Thus, comparator intensity ranged from standard care, wait-list, or no intervention to rehabilitation and active movement-based comparators, which may have influenced the magnitude and interpretation of between-group effects.

Outcome assessment was similarly heterogeneous. Overall autism symptom severity was assessed using the CARS, ABC, and ECA-R, whereas social communication/social responsiveness was assessed using the SCQ, SRS/SRS-2, and the ATEC social ability domain. Other studies examined more specific interpersonal constructs, including joint engagement, cooperative behavior, social closeness, interpersonal synchrony, emotion inference, and empathy. Motor outcomes included MABC-2 performance, gross motor development, and objective motor-coordination measures, while additional outcomes included gesture imitation, sensory and emotional functioning, verbal and non-verbal communication, and fNIRS-derived cortical activation. These measures represent related but non-equivalent constructs; accordingly, outcomes were quantitatively pooled only when they were judged sufficiently comparable within a prespecified domain, while more distinct outcomes were retained for narrative synthesis. The contribution of each included study to the quantitative and narrative syntheses is summarized in **Supplementary Table S1**.

### 3.2 Risk-of-bias assessment

Risk-of-bias assessments are summarized in **Figure 2**. Among the randomized studies assessed using RoB 2, most outcomes were judged as having some concerns. The outcomes reported by Lindor et al. were judged to be at high overall risk of bias, primarily because of concerns related to deviations from the intended intervention and missing outcome data (19). The remaining randomized studies were judged as having some concerns overall, most commonly because of limited reporting of the randomization process, outcome measurement, or selection of the reported result.

**Figure 2:**
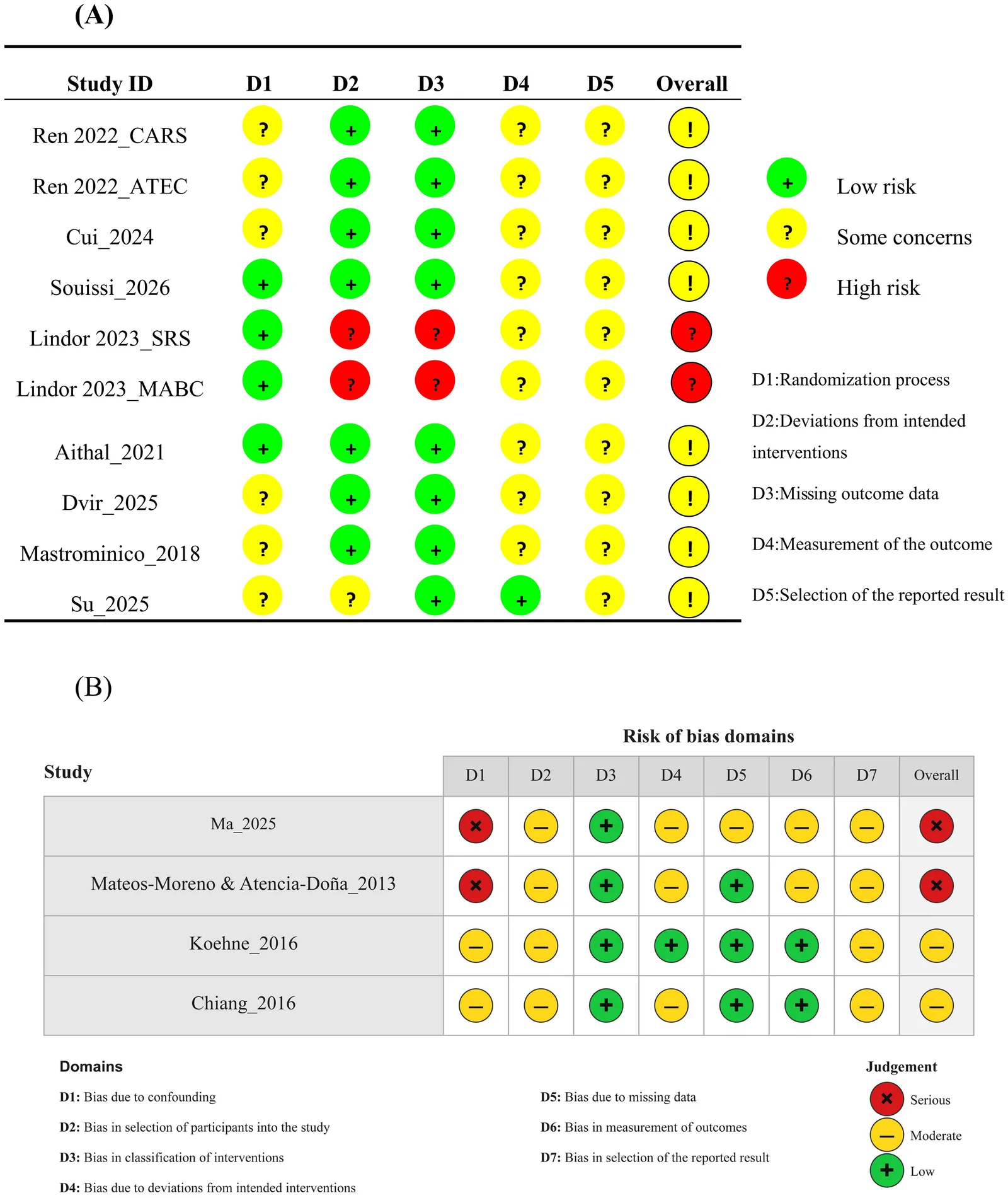
Risk-of-bias assessment of the included studies. **(A)** Risk of bias in randomized studies assessed using the Cochrane Risk of Bias 2 (RoB 2) tool. The five domains were: D1, bias arising from the randomization process; D2, bias due to deviations from intended interventions; D3, bias due to missing outcome data; D4, bias in measurement of the outcome; and D5, bias in selection of the reported result. Overall judgments were categorized as low risk, some concerns, or high risk. Separate rows were used for different outcomes from the same study when outcome-specific judgments differed. **(B)** Risk of bias in non-randomized controlled studies assessed using ROBINS-I. The seven domains were: D1, bias due to confounding; D2, bias in selection of participants into the study; D3, bias in classification of interventions; D4, bias due to deviations from intended interventions; D5, bias due to missing data; D6, bias in measurement of outcomes; and D7, bias in selection of the reported result. Overall judgments were made according to the ROBINS-I framework.

Among the four non-randomized controlled studies assessed using ROBINS-I, Ma et al. and Mateos-Moreno and Atencia-Doña were judged to be at serious overall risk of bias, with confounding representing the principal concern (29, 41). Koehne et al. and Chiang et al. were judged to be at moderate overall risk of bias. The domain-level judgments for both assessment tools are presented in **Figure 2**. Taken together, these judgments indicate that the evidence base was affected by methodological limitations across both randomized and non-randomized studies, which were considered when interpreting the quantitative and narrative findings. Accordingly, favorable findings from individual studies, particularly those derived from small trials or non-randomized designs, were interpreted as signals of potential benefit rather than confirmatory evidence of efficacy.

### 3.3 Overall autism symptom severity: narrative synthesis and individual randomized effect

Three studies (29, 41, 42) reported quantitative outcomes related to overall autism symptom severity, using the Autism Behavior Checklist (ABC) (43), Childhood Autism Rating Scale (CARS) (44, 45), and Revised Clinical Scale for the Evaluation of Autistic Behavior (ECA-R) (46). However, only Ren et al. was a randomized controlled trial. Ma et al. and Mateos-Moreno and Atencia-Doña were non-randomized controlled studies and were therefore not pooled with the randomized evidence.

In Ren et al., the post-intervention CARS score favored the intervention group, with an SMD of −3.09 (95% CI: −4.19 to −1.99). The underlying endpoint data were rechecked against the original report and confirmed to represent post-intervention means with SDs rather than SEs, change scores, or adjusted means. Because only one randomized study contributed to this outcome domain, no pooled meta-analysis was performed.

The two non-randomized studies also reported changes in overall symptom measures following dance- and movement-based interventions. These findings were retained in the narrative synthesis rather than quantitatively combined with the randomized trial because of differences in study design and outcome measurement. Given the different study designs, outcome instruments, and risk-of-bias profiles, the directionally favorable findings across these studies should not be interpreted as independent confirmation of a common treatment effect.

### 3.4 Social communication/social responsiveness: narrative synthesis and exploratory meta-analysis

Three studies (18, 19, 42) contributed data to the meta-analysis of social communication and social responsiveness. For the crossover study by Aithal et al., only first-period data were used. The analysis therefore included 36 participants in the intervention groups and 40 in the control groups. Outcomes were measured using the Social Communication Questionnaire (SCQ) (47), the Social Responsiveness Scale-2 Social Communication and Interaction (SRS-2 SCI) score (48), and the social ability domain of the Autism Treatment Evaluation Checklist (ATEC) (49). Ma et al. (29) also reported social-responsiveness outcomes but was not included in the primary meta-analysis because of its non-randomized design. Cui et al. assessed social responsiveness using the SRS; however, exact post-intervention group means and SDs were not reported in extractable numerical form. This outcome was therefore retained in the narrative synthesis rather than included in the social-function meta-analysis.

The random-effects meta-analysis yielded an SMD of −0.68 (95% CI: −1.58 to 0.23; *p* = 0.15), with substantial heterogeneity (I² = 72%) (**Figure 3**). The confidence interval crossed the line of no effect, and the pooled effect was not statistically significant. Negative SMDs favored the intervention because higher scores on the included measures represented greater social impairment.

**Figure 3:**
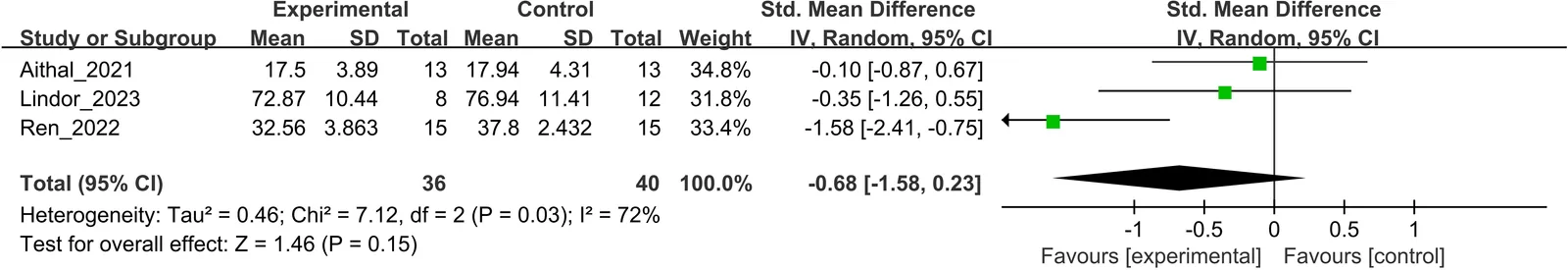
Random-effects meta-analysis of dance- and movement-based interventions on social communication and social responsiveness. Three randomized studies were included. For the crossover study by Aithal et al., only first-period data were used. Standardized mean differences (SMDs) with 95% confidence intervals (CIs) are shown. Negative SMDs favor the intervention because higher scores on the included measures indicate greater social impairment.

Although these measures were considered sufficiently related to permit exploratory pooling within a broad social communication/social responsiveness domain, they are not interchangeable measures of a single construct. The substantial heterogeneity observed across the three randomized studies may therefore reflect differences not only in intervention effects but also in outcome measurement, intervention content, participant characteristics, and comparator conditions. Because each instrument was represented by only one study, construct-specific subgroup analyses were not feasible. Accordingly, the pooled estimate should be interpreted as a broad summary of a heterogeneous evidence base rather than as a precise estimate of the effect of DMIs on a single social construct.

### 3.5 Motor function: narrative synthesis and exploratory meta-analysis

Three studies (19, 50, 51) contributed to the quantitative synthesis of motor function, comprising 27 participants in the intervention groups and 28 in the control groups. Motor outcomes were assessed using different measures, including the Movement Assessment Battery for Children-2 (MABC-2) (52), the Test of Gross Motor Development-3 (TGMD-3) (53), and an objective motor-coordination measure. SMDs were pooled using a random-effects model.

The pooled SMD was 2.04 (95% CI: −0.52 to 4.61; Z = 1.56, *p = 0.12*), with considerable heterogeneity (I² = 92%) (**Figure 4**). The confidence interval crossed zero, and the pooled effect was not statistically significant. Positive SMDs favored the intervention because higher scores represented better motor performance. The underlying endpoint data for Cui et al. and Souissi et al. were rechecked against the original reports and were confirmed to represent post-intervention means with SDs rather than SEs or change scores.

**Figure 4:**
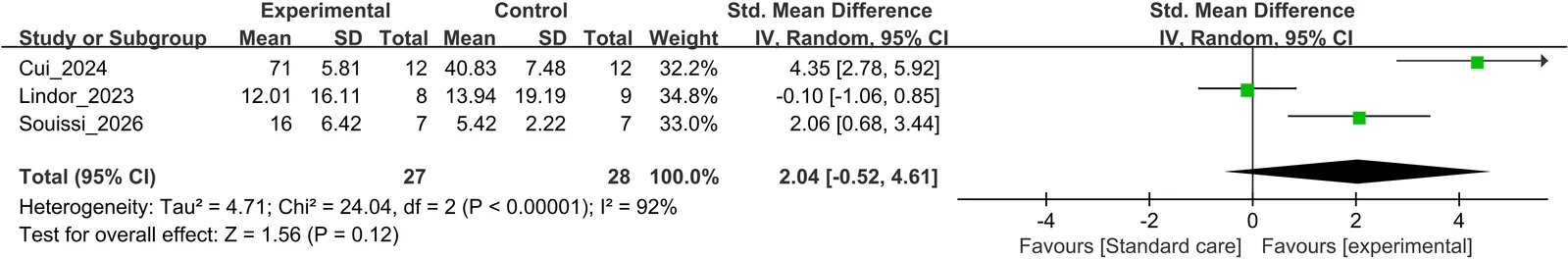
Random-effects meta-analysis of dance- and movement-based interventions on motor function. Three randomized studies were included. Standardized mean differences (SMDs) with 95% confidence intervals (CIs) are shown. Outcome directions were aligned so that higher scores represented better motor performance; therefore, positive SMDs favor the intervention.

Leave-one-out sensitivity analyses showed marked variation in the pooled estimate. Excluding Lindor et al. yielded an SMD of 3.17 (95% CI: 0.93 to 5.42; *p* = 0.005; I² = 78%). Excluding Souissi et al. yielded an SMD of 2.08 (95% CI: −2.28 to 6.44; *p* = 0.35; I² = 96%), whereas excluding Cui et al. yielded an SMD of 0.92 (95% CI: −1.20 to 3.04; *p* = 0.39; I² = 84%) (**Supplementary Figure S1**). Given that only three studies contributed to the analysis, these leave-one-out analyses were descriptive and should not be interpreted as establishing the robustness of the pooled effect.

The three studies also differed in intervention emphasis and motor-outcome definition, ranging from broader dance-based or psychomotor programs to measures of gross motor performance, MABC-2 performance, and objective motor coordination. Together with the very small study-level samples and considerable statistical heterogeneity, these differences limit the interpretability of the pooled SMD. The meta-analytic estimate should therefore be considered exploratory and should not be interpreted as evidence of a uniform motor effect across DMI formats.

### 3.6 Other outcomes: narrative synthesis

Several additional outcomes were not quantitatively pooled because the available measures represented distinct constructs or were reported by only one or two studies. These included joint engagement, empathy and emotion inference, cooperative behavior, interpersonal synchrony, gesture imitation, sensory and emotional outcomes, verbal/non-verbal communication, and neurophysiological measures (**Table 1**).

Chiang et al. reported greater child-initiated supported and coordinated joint engagement in the intervention group at the 3-month follow-up (54). Dvir et al. reported greater cooperative behavior at follow-up and greater social closeness with familiar peers after synchronized movement, whereas no clear between-group difference was observed for work-related stress. Koehne et al. reported improvement in emotion inference and movement-related synchronization outcomes, while empathic feelings did not show a corresponding improvement. Mastrominico et al. found no significant overall intervention effect on empathy.

Souissi et al. reported higher post-intervention scores for simple hand and arm gesture imitation in the intervention group, whereas post-intervention IQ did not differ significantly between groups. Su et al. reported changes in interpersonal synchrony and fNIRS-derived cortical activation following creative movement. Other sensory, emotional, language, and communication outcomes reported by Ma et al., Mateos-Moreno and Atencia-Doña, and Aithal et al. were retained as narrative evidence because they were not sufficiently comparable for quantitative synthesis.

Cui et al. reported significant post-intervention between-group differences in the SRS total score and its social domains following dance therapy, but the exact post-intervention means and SDs required for effect-size calculation were not available in numerical form. The SRS findings were therefore interpreted narratively. Taken together, these study-specific findings suggest that potential effects may be more apparent for selected domains of interpersonal coordination, engagement, or motor behavior than for broader constructs such as empathy or global autism-related functioning. However, because these outcomes were generally assessed in single small studies using different instruments and intervention formats, the apparent domain-specific pattern remains hypothesis-generating rather than confirmatory.

### 3.7 Certainty of evidence

The GRADE assessment is summarized in **Table 2**. The certainty of evidence for social communication/social responsiveness was rated as very low. Evidence was downgraded for risk of bias because one contributing randomized trial was judged to be at high risk of bias while the remaining trials had some concerns, for inconsistency because substantial heterogeneity was present (I² = 72%), and for imprecision because the 95% CI was wide and crossed the line of no effect. The certainty of evidence for motor function was also rated as very low because of serious risk of bias, considerable inconsistency (I² = 92%), and imprecision associated with the small sample size and wide confidence interval. For overall autism symptom severity, the certainty of evidence was rated as low. The evidence was based on a single small randomized trial with some concerns for risk of bias and was downgraded for risk of bias and imprecision (35) (**Table 2**).

**Table 2:** GRADE Summary of Findings for the main outcome domains.

Certainty assessment							№ of patients		Effect		Certainty	Importance
№ of studies	Study design	Risk of bias	Inconsistency	Indirectness	Imprecision	Other considerations	Dance- and movement-based interventions	Control conditions	Relative (95% CI)	Absolute (95% CI)		
Social communication												
3	randomised trials	serious^a^	serious^b^	not serious	serious^c^	none	36	40	–	SMD **0.68 SD lower** (1.58 lower to 0.23 higher)	⨁◯◯◯ Very low^a,b,c^	CRITICAL
Motor function												
3	randomised trials	serious^d^	serious^e^	not serious	serious^f^	none	27	28	–	SMD **2.04 higher** (0.52 lower to 4.61 higher)	⨁◯◯◯ Very low^d,e,f^	CRITICAL
Overall autism symptom severity												
1	randomised trials	serious^g^	not serious	not serious	serious^h^	none	15	15	–	SMD **3.09 SD lower** (4.19 lower to 1.99 lower)	⨁⨁◯◯ Low^g,h^	CRITICAL

Question: Dance- and movement-based interventions compared with control conditions for autism spectrum disorder.

Setting: Clinical, educational, and community settings.

CI, confidence interval; SMD, standardised mean difference.

Explanations.

^a^Downgraded one level because one contributing randomized trial (Lindor et al.) was judged to be at high risk of bias, while the remaining contributing trials had some concerns.

^b^Substantial heterogeneity was observed across the three RCTs (I² = 72%), indicating variability in the magnitude of the intervention effects.

^c^The 95% CI was wide and crossed the line of no effect (SMD −0.68, 95% CI −1.58 to 0.23), with a relatively small total sample size (n = 76).

^d^Downgraded one level because Lindor et al. was judged to be at high risk of bias for the motor outcome, while the other contributing randomized trials had some concerns.

^e^Considerable heterogeneity was observed across the three RCTs (I² = 92%), with substantial variation in the magnitude of the intervention effects.

^f^The total sample size was small (n = 55), and the 95% CI was wide and crossed the line of no effect (SMD 2.04, 95% CI −0.52 to 4.61).

^g^Downgraded one level because the evidence was based on a single randomized trial that was judged to have some concerns in the RoB 2 assessment.

^h^The evidence was based on a single small RCT with only 30 participants, resulting in limited precision despite the observed effect.

Certainty of evidence was assessed for social communication/social responsiveness, motor function, and overall autism symptom severity using the GRADE framework. CI, confidence interval; SMD, standardized mean difference; GRADE, Grading of Recommendations Assessment, Development and Evaluation. Bold text indicates serious limitations in GRADE assessment domains that contributed to downgrading the certainty of evidence.

## 4 Discussion

This systematic review identified a small and clinically heterogeneous evidence base spanning multiple DMI formats, comparator conditions, age groups, and outcome constructs. Accordingly, the structured narrative synthesis provides the primary framework for interpretation, whereas the meta-analyses should be regarded as exploratory summaries of selected randomized evidence. After separating randomized from non-randomized evidence, pooled effects for social communication/social responsiveness and motor function were not statistically significant and were rated as very low-certainty evidence. For overall autism symptom severity, only one randomized trial was available, resulting in low-certainty evidence and precluding pooling. Narrative findings suggested potentially favorable changes in selected interpersonal and functional outcomes, but the magnitude, consistency, and generalizability of these signals remain uncertain.

Previous quantitative evidence has suggested that dance-based interventions may reduce overall autism-related symptom severity. Chen et al. reported a favorable pooled effect in a meta-analysis of controlled studies. However, the present review separated randomized and non-randomized evidence rather than combining different study designs in the same primary meta-analysis. Under this approach, only Ren et al. contributed randomized evidence for overall autism symptom severity, whereas Ma et al. and Mateos-Moreno and Atencia-Doña were retained in the narrative synthesis because of their non-randomized designs. Although the randomized trial showed a marked between-group difference in CARS scores, it included only 30 participants. The GRADE assessment therefore indicated low-certainty evidence, with downgrading for risk of bias and imprecision related to the single small trial. Consequently, the current evidence is insufficient to determine whether the observed effect is reproducible across populations, intervention formats, and measures of overall autism symptom severity.

Social communication and social responsiveness showed a more uncertain pattern than suggested by the original mixed-design analysis. When the primary meta-analysis was restricted to randomized studies, the pooled estimate remained in the intervention-favoring direction but was not statistically significant, and the certainty of evidence was rated as very low. This differs in strength from earlier meta-analyses that reported favorable effects of dance-based or related movement interventions on social outcomes (25, 27). Broader physical-activity reviews have also reported improvements in social communication and sociability in children and adolescents with ASD, although these reviews included intervention modalities that were not directly comparable with DMIs. Differences in the populations, comparator conditions, intervention content, and outcome definitions may partly explain the variation across reviews.

An additional source of uncertainty was the conceptual diversity of the measures included in the present social analysis. The SCQ, SRS-2 Social Communication and Interaction score, and ATEC social ability domain all capture aspects of social or communicative functioning, but they are not interchangeable measures of a single construct. The substantial heterogeneity observed across the three randomized studies may therefore reflect not only differences in intervention effects but also differences in what was being measured. The available evidence was therefore insufficient for construct-level comparisons.

Several features of DMIs provide plausible explanations for the direction of the social findings, although the present evidence cannot establish a specific therapeutic mechanism. Interpersonal synchrony has been described as atypical in ASD and has been proposed as a potential target for social intervention (10). Dance- and movement-based programs commonly incorporate imitation, mirroring, rhythm, and shared movement, requiring participants to attend to another person’s actions and coordinate their own behavior within an interpersonal context. Findings from individual studies were compatible with this hypothesis: Koehne et al. reported changes in synchronization, movement reciprocity, and emotion inference, while Dvir et al. reported greater cooperative behavior following synchronized group movement. However, empathy outcomes were less consistent, and an earlier meta-analysis similarly found little evidence of improvement in empathy (25). Improvements in observable coordination or social engagement should therefore not be assumed to represent broader changes in empathic functioning.

Motor function warrants particular attention because motor coordination, balance, motor planning, and related difficulties are frequently reported in individuals with ASD (6–8). In the present review, the pooled estimate was in the intervention-favoring direction but did not reach statistical significance, and the certainty of evidence was rated as very low. Considerable heterogeneity was observed across the three randomized studies, and the leave-one-out analyses showed that the pooled estimate was sensitive to the inclusion of individual studies. In particular, although exclusion of Lindor et al. (19) resulted in a statistically significant pooled estimate, substantial heterogeneity remained, whereas exclusion of either Cui et al. (50) or Souissi et al. (51) produced estimates with confidence intervals crossing the line of no effect. The motor findings therefore cannot be regarded as robust across study combinations.

The variability in motor effects may partly reflect differences in both intervention content and outcome measurement. The three studies assessed different aspects of motor performance, including gross motor skills, MABC-2 performance, and objective motor coordination, while intervention duration and therapeutic emphasis also differed considerably. The particularly large study-level effects reported by Cui et al. and Souissi et al. were rechecked against the original reports; the values used in the meta-analysis were post-intervention endpoint means with SDs rather than SEs or change scores. These large effects therefore did not appear to result from an extraction or variance-conversion error, but both studies involved small samples, which limits the precision and stability of their effect estimates.

The uncertain pooled motor finding should not be interpreted as evidence that structured movement interventions have no relevance to motor development in ASD. Evidence from the broader exercise literature has generally been more favorable. Recent systematic reviews and meta-analyses have reported improvements in motor and related functional outcomes following structured physical exercise or motor interventions in children with ASD (55–57). However, these findings cannot be directly extrapolated to DMIs because exercise and motor-training programs differ from DMT and related dance-based approaches in therapeutic rationale, intervention components, intensity, and intended outcomes. The contrast nevertheless highlights the need for future DMI trials to include validated motor measures as prespecified outcomes and to report intervention dose and motor-specific treatment components in sufficient detail.

The outcomes that could not be quantitatively pooled further illustrate the breadth and conceptual heterogeneity of the evidence base. Individual studies reported changes in joint engagement, cooperative behavior, interpersonal synchrony, emotion inference, gesture imitation, and movement reciprocity, but these constructs were assessed using substantially different methods. Chiang et al. (54) reported changes in joint engagement following a caregiver-mediated intervention incorporating creative body-movement play, while Koehne et al. and Dvir et al. examined interpersonal synchronization and related social outcomes. These findings indicate that movement-based interventions may influence specific forms of interpersonal engagement, but they should not be treated as interchangeable measures of a common social construct.

Empathy findings were less consistent. Mastrominico et al. (58) did not demonstrate a clear overall intervention effect on empathy, and an earlier meta-analysis similarly reported little evidence of improvement in this domain (25). Su et al. (59) combined behavioral measures of interpersonal synchrony with functional near-infrared spectroscopy (fNIRS) and reported intervention-related differences in cortical activation following creative movement. Such neurophysiological findings are of interest for hypothesis generation but remain preliminary and should not be interpreted as evidence of an established neural mechanism. More generally, the small number of studies assessing each of these outcomes and the substantial differences in measurement methods prevented defensible quantitative synthesis.

The breadth of the DMI construct remains an important interpretive consideration. Although all included programs used structured dance or movement as a central component, they differed substantially in therapeutic framework, delivery format, dose, comparator condition, and intended target. The pooled estimates should therefore not be generalized uniformly across all DMI formats. Future trials should report intervention components, therapist qualifications, theoretical framework, dose, and treatment fidelity more consistently to distinguish formal DMT/DMP from other structured movement-based approaches.

Taken together, the present findings support a cautious interpretation of DMIs as potential complementary approaches rather than established stand-alone treatments for ASD. The randomized evidence for social communication/social responsiveness and motor function remained uncertain and was rated as very low certainty, while the evidence for overall autism symptom severity was based on a single small, randomized trial and was rated as low certainty. The current evidence therefore does not establish that DMIs are superior to other physical, behavioral, or creative interventions, nor does it identify an optimal intervention format, intensity, frequency, or duration. Their potential value may lie in combining structured movement with opportunities for interpersonal coordination and non-verbal engagement, but larger and methodologically rigorous trials are required to determine whether these features translate into reproducible clinical benefits.

## 5 Limitations

Several limitations should be considered when interpreting the findings. The number of studies contributing to each quantitative synthesis was small, and sample sizes were generally modest, which limited the precision of the pooled estimates and the feasibility of subgroup analyses. In addition, the included studies varied in participant characteristics, intervention formats, comparator conditions, and outcome measures, contributing to heterogeneity across analyses. Some methodological limitations were also identified in the primary studies, particularly in relation to incomplete reporting and the use of subjective outcome measures in some trials. In addition, Chinese-specific databases such as CNKI, Wanfang, and VIP were not searched, and therefore some locally indexed Chinese studies captured by previous reviews may not have been identified. Although the structured narrative synthesis helps clarify these sources of variation, it does not eliminate the limitations of the underlying evidence.

Despite these limitations, the present review strengthened the synthesis by separating randomized and non-randomized evidence, applying design-appropriate risk-of-bias tools, conducting sensitivity analyses, and formally assessing the certainty of evidence using GRADE. Future studies with larger samples, more standardized intervention reporting, and more consistent outcome measures will help clarify the magnitude and clinical relevance of the observed effects.

## 6 Conclusion

This systematic review identified a limited and heterogeneous evidence base for dance- and movement-based interventions across age groups. The structured narrative synthesis suggested potentially favorable signals in several domains, particularly interpersonal engagement and coordination, but these findings were derived largely from small studies using heterogeneous interventions and outcome measures. Exploratory RCT-only meta-analyses for social communication/social responsiveness and motor function were not statistically significant and were rated as very low-certainty evidence, while evidence for overall autism symptom severity was limited to a single small, randomized trial and was rated as low certainty. DMIs may have potential as complementary interventions for ASD, but current evidence is insufficient to establish consistent efficacy, identify an optimal intervention format, or determine which participants are most likely to benefit. Larger, methodologically rigorous randomized trials with standardized intervention descriptions and outcome measures are needed to clarify their clinical role.

## Data Availability

The original contributions presented in the study are included in the article/**Supplementary Material**. Further inquiries can be directed to the corresponding author.

## Supplementary material

Supplementary Figure 1 Leave-one-out sensitivity analyses for the motor-function meta-analysis. Each panel shows the random-effects pooled estimate after sequential exclusion of one study: (A) Lindor et al.; (B) Souissi et al.; and (C) Cui et al. The analyses were conducted to examine the influence of individual studies on the pooled motor-function estimate.

Supplementary Table 1 Study-to-synthesis mapping of the 12 included studies. The table summarizes the contribution of each included study to the quantitative and narrative syntheses across overall autism symptom severity, social communication/social responsiveness, motor function, and other outcomes.

Supplementary Table 2 Extracted quantitative data used in the exploratory quantitative syntheses. The values for Ren et al. (2022), Cui et al. (2024), and Souissi et al. (2026), which generated particularly large, standardized effect estimates, were rechecked against the original reports and confirmed to represent endpoint means with SDs rather than SEs, change scores, or adjusted means.
